# Polar or Charged Residues Located in Four Highly Conserved Motifs Play a Vital Role in the Function or pH Response of a UPF0118 Family Na^+^(Li^+^)/H^+^ Antiporter

**DOI:** 10.3389/fmicb.2020.00841

**Published:** 2020-05-07

**Authors:** Lidan Wang, Qiao Zou, Mingxue Yan, Yuting Wang, Sijia Guo, Rui Zhang, Yang Song, Xiaofang Li, Huiwen Chen, Li Shao, Lin Meng, Juquan Jiang

**Affiliations:** Department of Microbiology and Biotechnology, College of Biological Sciences, Northeast Agricultural University, Harbin, China

**Keywords:** UPF0118 family, Na^+^(Li^+^)/H^+^ antiporter, functional motif, pH response, Na^+^(Li^+^) translocation

## Abstract

Functionally uncharacterized UPF0118 family has been re-designated as autoinducer-2 exporter (AI-2E) family since one of its members, *Escherichia coli* YdgG, was identified to function as an AI-2E. However, it’s very likely that AI-2E family members may exhibit significantly distinct functions due to low identities between them. Recently, we identified one member of this family designated as UPF0118 to represent a novel class of Na^+^(Li^+^)/H^+^ antiporters. In this study, we presented that UPF0118, together with its homologs, should represent an independent group of AI-2E family, designated as Na^+^/H^+^ Antiporter Group. Notably, this group shows five highly conserved motifs designated as Motifs A to E, which are not detected in the majority of AI-2E family members. Functional analysis established that polar or charged residues located in Motif A to D play a vital role in Na^+^(Li^+^)/H^+^ antiport activity or pH response of UPF0118. However, three basic residues located in Motif E are not involved in the function of UPF0118, although the truncation of C terminus resulted in the non-expression of this transporter. Therefore, we propose that E_179_-R_182_-K_215_-Q_217_-D_251_-R_292_-R_293_-E_296_-K_298_-S_30__7_ located in Motifs A to D can be used for signature functional motifs to recognize whether AI-2E family members function as Na^+^(Li^+^)/H^+^ antiporters. Current findings positively contribute to the knowledge of molecular mechanism of Na^+^, Li^+^ transporting and pH response of UPF0118, and the functional prediction of uncharacterized AI-2E family members.

## Introduction

Na^+^/H^+^ antiporters are a category of secondary transmembrane proteins which are widely distributed in cytoplasmic or organelle membranes of almost living organisms ranging from bacteria to human. These antiporters play vital roles in not only mediating the circulation of various monovalent cations, mainly Na^+^ and K^+^, even Ca^2^^+^, inside and outside the cells and maintaining intracellular pH homeostasis, but also participating in many biological process including signal molecule transmission, spore development, antibiotic release, cell volume control and etc. (Padan and Schuldiner, 1994; Padan et al., 2005; Padan and Landau, 2016; Uzdavinys et al., 2017; Masrati et al., 2018). As one of most prevalent physiological strategies, bacteria employ Na^+^/H^+^ antiporters to adapt themselves to high saline-alkaline environment during their survival and growth (Padan et al., 2001; Krulwich et al., 2009). In the Transporter Classification Database (TCDB) system (Saier et al., 2016), bacterial Na^+^/H^+^ antiporters have been re-classified into ten major families or superfamilies based on the phylogenetic relationship: (1) monovalent cation/proton antiporter-1 (CPA-1) family, such as NhaG (Gouda et al., 2001), NhaH (Yang et al., 2006), NhaP (Utsugi et al., 1998), etc; (2) CPA-2 family, such as GerN (Southworth et al., 2001) and NapA (Waser et al., 1992); (3) CPA-3 family mainly including six- or seven-subunit Mrp systems (Ito et al., 1999, 2000, 2001; Swartz et al., 2009; Meng et al., 2014; Cheng et al., 2016; Xu et al., 2018); (4) major facilitator superfamily (MFS) including Tet(L) (Cheng et al., 1994), MdfA (Lewinson et al., 2004), MdtM (Holdsworth and Law, 2013), and MdrP (Abdel-Motaal et al., 2018); (5) Ca^2^^+^:H^+^ antiporter-2 (CaCA2) family, such as ChaA (Ivey et al., 1993); (6) NhaA family (Karpel et al., 1988); (7) NhaB family (Pinner et al., 1992); (8) NhaC family including NhaC (Ito et al., 1997) and MleN (Wei et al., 2000); (9) NhaD family (Nozaki et al., 1998; Liu et al., 2005; Zhang et al., 2014; Cui et al., 2016; Wang et al., 2017; Yang et al., 2018); (10) NhaE family (Sousa et al., 2013). The latter five families [families (6)–(10)] were formerly classified to CPA-1 family in the TCDB system. Also, NhaA was classified into CPA-2 family, such as in the study by Masrati et al. (2018), and etc. Most non-halophilic bacteria were predicted to contain 5–9 distinct Na^+^/H^+^ antiporters belonging to the above-mentioned families or superfamilies. In contrast, halophilic bacteria have been widely accepted to possess a larger number of Na^+^/H^+^ antiporters for the adaptation under high saline-alkaline stress (Ventosa et al., 1998; Oren, 1999; Krulwich et al., 2009). To increase the number of Na^+^/H^+^ antiporters, we speculate that halophilic bacteria may be forced to evolve more families of transporters into novel Na^+^/H^+^ antiporters or transporters with Na^+^/H^+^ antiport activity. That was supported by our reports that several novel transporters, PsmrAB, UPF0118, UmpAB, RDD, and MceT, have been successively cloned from different slightly or moderately halophilic bacteria and identified to function as Na^+^/H^+^ antiporters or possess Na^+^/H^+^ antiport activity (Jiang et al., 2013a; Dong et al., 2017; Meng Y. et al., 2017; Shao et al., 2018; Xu et al., 2019). For example, UPF0118, UmpAB, and RDD formerly belonged to three functionally unknown families, autoinducer-2 exporter (AI-2E) family (Dong et al., 2017), DUF1538 family (Meng Y. et al., 2017), and RDD family (Shao et al., 2018), respectively. PsmrAB and MceT belongs to two known families with other protein functions, paired small multidrug resistance protein (PSMR) family (Jiang et al., 2013a) and cation diffusion facilitator (CDF) family (Xu et al., 2019), respectively. Due to belonging to the formerly uncharacterized families or superfamilies, investigation of these novel Na^+^/H^+^ antiporters may provide new insights into Na^+^/H^+^ antiport molecular mechanisms. Also, novel Na^+^/H^+^ antiporters have no homologs in the non-halophilic nitrogen-fixing, growth-promoting or biologically controlling microorganisms, or even many plants especially crops. Therefore, these novel Na^+^/H^+^ antiporter genes are more likely to successfully improve the saline-alkaline resistance of gene-engineered microorganisms or transgenic plants.

In our previous study, a UPF0118 family (currently named AI-2E family) transporter from the moderately halophilic bacterium *Halobacillus andaensis* has been identified to represent a novel class of Na^+^(Li^+^)/H^+^ antiporters (Dong et al., 2017). Hereby, we still use this designation due to its functional difference from other AI-2E family members. In the TCDB system, there are two major categories of AI-2E family members with the TC numbers from 2.A.86.1.1 to 2.A.86.1.16 (2.A.86.1.15 for UPF0118) and from 2.A.86.2.1 to 2.A.86.2.3, respectively (Saier et al., 2016). Although these members are classified into AI-2E family, identities between them are quite low. For example, there are three AI-2E family members, YtvI, YueF, and YrrI, in the genome of non-halophilic *Bacillus subtilis* subsp. *subtilis* strain 168. However, these three members exhibit quite low identities at about 20% between them. Also, the genome of moderately halophilic *H. andaensis* has been sequenced recently by our lab. As a result, we found that there are six AI-2E family members including UPF0118 with significantly low identities ranging from 15 to 21% in the genome of *H. andaensis* (Data unpublished). Therefore, we speculate that AI-2E family members may exhibit a significant difference in function due to low identities between them, although these members are temporarily categorized into AI-2E family.

Interestingly, UPF0118 and its representative homologs share five fully conserved motifs even at a range of 58–82% identities (Dong et al., 2017). However, these conserved motifs are not detected in the majority of the members collected in the TCDB system (Saier et al., 2016). Therefore, we hypothesize that these five motifs designated as Motifs A to E may be used to differentiate UPF0118 and its homologs from other AI-2E family members. In order to address the above hypothesis and also explore molecular mechanism of UPF0118 as a Na^+^(Li^+^)/H^+^ antiporter, we first analyze the phylogenetic relationship between UPF0118 and its homologs and AI-2E members collected in the TCDB system. Also, we further discover the roles of polar or charged amino acid residues located in the above five motifs of UPF0118 via site directed mutagenesis. Consequently, we found out that UPF0118 and its homologs should represent an independent group designated as Na^+^/H^+^ Antiporter Group. More importantly, we propose that E_179_-R_182_-K_215_-Q_217_-D_251_-R_292_-R_293_-E_296_-K_298_-S_30__7_ located in Motifs A to D can be used for signature functional motifs to recognize whether AI-2E family members function as Na^+^(Li^+^)/H^+^ antiporters. These findings positively contribute to the understanding of molecular mechanism of Na^+^, Li^+^ transporting and pH response of UPF0118. AI-2E family includes a large number of uncharacterized members except for *Escherichia coli* YdgG and *H. andaensis* UPF0118 (Herzberg et al., 2006; Dong et al., 2017). Therefore, current findings will also be helpful to recognize whether uncharacterized AI-2E family members may function as Na^+^/H^+^ antiporters.

## Materials and Methods

### Strains, Plasmids, and Growth Conditions

Supplementary Table S1 shows the strains and plasmids used in this study. The transformants of a three-major-Na^+^/H^+^ antiporter-deficient *E. coli* mutant KNabc (Δ*nhaA*Δ*nhaB*Δ*chaA*) (Nozaki et al., 1996) were grown in the LBK medium with the composition of 1.0% tryptone, 0.5% yeast extract, and 87 mM KCl as described previously (Karpel et al., 1988). Growth tests for salt-tolerance and alkaline pH resistance were performed as described in our recent studies (Dong et al., 2017; Meng Y. et al., 2017; Abdel-Motaal et al., 2018; Shao et al., 2018; Xu et al., 2019). Briefly, 1% overnight cultures of *E. coli* KNabc transformants grown at 37°C in the LBK medium at pH 7.0 were innoculated into fresh LBK medium at pH 7.0, and then the growth tests were carried out in the LBK media containing the indicated concentrations of NaCl or LiCl, or at the indicated pH plus 50 mM NaCl (A, right panel). Growth was ended on 24 h and then OD_600*nm*_ was evaluated. The *phoA*-deficient *E. coli* mutant DH5α was used as a host strain to test the orientation of UPF0118 in the cytoplasmic membranes as described previously (Meng L. et al., 2017). Ampicillin with the final concentration at 50 μg/ml was used for the selection and growth of *E. coli* transformants.

### Bioinformatic Analyses

A neighbor-joining phylogenetic tree was constructed with a bootstrap analysis (1000 replications) for the stability of clusters (Saitou and Nei, 1987). Protein alignment was performed by using BlastP at the National Center for Biotechnology Information (NCBI) website https://blast.ncbi.nlm.nih.gov/Blast.cgi?PROGRAM=blastp&PAGE_TYPE=BlastSearch&LINK_LOC=blasthome. Topological analysis was carried out via the multiple web-based softwares including HMMTOP, TMHMM, Tmpred, PredTMR, SOSUI, Phyre 2, and PredictProtein. Weblogo was created at the website http://weblogo.threeplusone.com/create.cgi.

### PhoA Activity Assay

UPF0118 fusions with signal peptide-less PhoA at the N or C termini were constructed under the control of native promoter of *upf0118* using the primers listed in Supplementary Table S1 via a routine overlapping PCR technique as described previously (Meng L. et al., 2017), and checked by sequencing at Beijing Genomics Institute (Beijing, China), and then transformed into the *phoA*-deficient *E. coli* mutant DH5α. Also, *E. coli* mutant DH5α transformed with the empty vector or expressing UPF0118 alone was used as the negative controls. The transformants were grown to observe the lawn color on the LB medium plate containing 0.4 mg/ml 5-bromo-4-chloro-3-indolylphosphate as the substrate, as described previously (Meng L. et al., 2017). The alkaline phosphatase activities of the above-mentioned *E. coli* DH5α transformants were analyzed as described previously (Rothman et al., 1996), and expressed as U/OD_600*nm*_.

### Construction of Site-Directed or C Terminus-Truncated Variants of UPF0118

All the site-directed or C terminus-truncated variants of UPF0118 were constructed using pET22b-P-UPF0118 as a template and the corresponding primers listed in Supplementary Table S1 via a Fast Mutagenesis System kit purchased from TransGen Biotech Co., Ltd. (Beijing, China), as described in our recent studies (Shao et al., 2018; Xu et al., 2019). All the final UPF0118 variants were re-sequenced to confirm the accuracy of mutagenesis, and the corresponding plasmids were transformed into *E. coli* KNabc for growth tests and Na^+^(Li^+^)/H^+^ antiport activity assays.

### Preparation of Everted Membrane Vesicles

Everted membrane vesicles were prepared from *E. coli* KNabc transformants by the French press method as described in our recent studies (Dong et al., 2017; Meng Y. et al., 2017; Abdel-Motaal et al., 2018; Shao et al., 2018; Xu et al., 2019). Cells of *E. coli* KNabc transformants carrying UPF0118 or its variants, or the empty vector were cultured in LBK media to exponential phase. Cells were harvested and re-suspended in a buffer containing 10 mM Hepes-Tris (pH 7.0), 140 mM choline chloride, 0.5 mM dithiothreitol and 250 mM sucrose, and broken by one passage through a JG-1A French Press (NingBo Scientz Biotechnology Co., Ltd., China) at 2000 psi system pressure. Cell debris was removed by centrifugation at 5000 × *g*, 4°C for 10 min, and the supernatant was centrifuged at 100,000 × *g* for 1 h. Everted membrane vesicles were finally separated from the supernatant and re-suspended in the same buffer as above, and stored at -80°C for the following Na^+^(Li^+^)/H^+^ antiport assay and protein expression level analysis.

### Protein Expression Assay by Western Blot

SDS-PAGE and western blots were performed as described in our recent studies (Dong et al., 2017; Meng Y. et al., 2017; Abdel-Motaal et al., 2018; Shao et al., 2018; Xu et al., 2019). The everted membrane vesicles equivalent to 100 μg of total membrane protein were subjected to SDS-PAGE and western blot analysis. His_6_-tag labeled proteins were detected using a rabbit anti-His_6_ tag antibody (Beyotime Biotechnology Co., Ltd., Shanghai, China) and a goat anti-rabbit horseradish peroxidase labeled secondary antibody (Nachuan Biotechnology Co., Ltd., Changchun, China). Western blots were visualized by using a BeyoECL Star kit (Beyotime Biotechnology Co., Ltd., Shanghai, China) via a Tannon-5200 multi chemiluminescent imaging system (Tanon Co., Ltd., China).

### Na^+^(Li^+^)/H^+^ Antiport Assay

Na^+^(Li^+^)/H^+^ antiport activities were measured by using an acridine orange fluorescence dequenching protocol, as described in our recent studies (Dong et al., 2017; Meng Y. et al., 2017; Abdel-Motaal et al., 2018; Shao et al., 2018; Xu et al., 2019). A reaction mixture includes 140 mM choline chloride, 5 mM Mg_2_SO_4_, 1 μM acridine orange, with the pH of the mixture to 6.5 to 9.5 adjusted with a 10 mM BTP (Bis-Tris Propane) buffer. Respiration-dependent formation of ΔpH was initiated by the addition of 10 mM Tris-D-lactate. Na^+^(Li^+^)/H^+^ antiport activity was estimated based on its ability to dissipate the established ΔpH upon addition of final concentrations at 10 mM NaCl or LiCl. The antiport activities were expressed as the percentage ratio of dequenched fluorescence by NaCl or LiCl to the lactate-induced fluorescence quenching. The acridine orange fluorescence was monitored with excitation wavelength at 492 nm and emission wavelength at 526 nm using a Hitachi F-7000 fluorescence spectrophotometer (Hitachi Ltd., Tokyo, Japan). *K*_0_._5_ values of wild-type UPF0118 or its variants for the transported cations were calculated by plotting Na^+^/H^+^ and Li^+^/H^+^ antiport activity as the respective functions of cation concentrations, followed by a non-linear regression analysis obtained with Prism 7.0, as described in our previous study (Dong et al., 2017).

## Results

### Topological Prediction of UPF0118 and Orientation of N and C Termini by PhoA Assay

**FIGURE 1:**
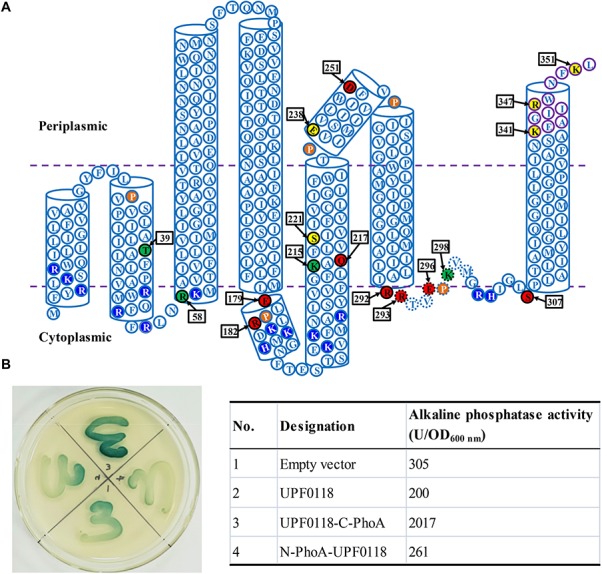
Topological model of *Halobacillus andaensis* UPF0118. Topological model **(A)** was predicted based on the topological analysis using the deduced amino acid sequence of *H. andaensis* UPF0118 via the multiple web-based softwares including HMMTOP, TMHMM, Tmpred, PredTMR, SOSUI, and Phyre 2 (Supplementary Table S2) and PredictProtein (Supplementary Table S3). Six predicted transmembrane helices and two predicted periplasm- or cytoplasm-exposed helices are shown in open cylinders. Membrane-buried residues in Loop5-6 are shown in dotted-line-border circles. Conserved charged or polar amino acid residues between *H. andaensis* UPF0118 and its selected homologs are highlighted in black with black-border circles. Functionally important residues are shown in red filled circles, pH response-related residues in green filled circles, and other functionally unrelated residues in yellow filled circles. Five proline residues possibly resulting in the broken helices and basic residues controlling the positive-inside rule are highlighted in brown filled circles and blue filled circles, respectively. The orientation of N and C termini were determined by the PhoA assay on the LB medium plate containing 5-bromo-4-chloro-3-indolylphosphate as the substrate [**(B)**, left panel] and alkaline phosphatase activity assay [**(B)**, right table].

In our previous study, we performed the topological analysis of UPF0118 using a web-based software TopPred II and found that UPF0118 was predicted to contain six transmembrane segments (TMSs, sometimes designated as transmembrane regions) (Dong et al., 2017). However, this transmembrane protein was predicted to contain seven TMSs and cover the different amino acid residue ranges even for TMSs by the software HMMTOP enclosed in the TCDB system (Supplementary Table S2). To better clarify the number of transmembrane helices (TMHs) of UPF0118 and the residue ranges of its transmembrane regions, we re-carried out the topological analysis of UPF0118 via the multiple web-based softwares including HMMTOP, TMHMM, TMpred, PredTMR, SOSUI, and Phyre 2 (Supplementary Table S2) and PredictProtein (Supplementary Table S3). The former six softwares predicted UPF0118 to contain 7–8 TMHs, with one exception of SOSUI showing six TMHs (Supplementary Table S2). Also, TMHMM, TMpred, and Phyre 2 predicted the orientation of N and C termini of UPF0118 (Supplementary Table S2). In contrast, PredictProtein predicted UPF0118 to contain eight helices with more detailed parameters including helix region, TMH region and buried region (Supplementary Table S3). For example, this software showed that UPF0118 contains eight helices including six TMHs and two non-transmembrane helices (α helix 1: 180 – 190; α helix 2: 238 – 252). Interestingly, TMH6 (308 – 348) covers two transmembrane regions (308 – 326 and 328 – 346) (Supplementary Table S3). Notably, one loop designated as Buried Loop5-6 (292 – 300) is located between TMH5 and TMH6 and buried in the cytoplasmic membranes (Supplementary Table S3). Pro (P) residues of transmembrane proteins can kink α helices or result in the discontinuous α helices by interrupting the hydrogen bonding network between Pro and its No. −4 residue (Deber and Therien, 2002). Also, charge distribution of transmembrane proteins is usually under the control of “positive inside rule,” presumably due to the interaction of basic residues with the polar head groups of lipid molecules (Jiang D. et al., 2013). Finally, a topological model of UPF0118 was predicted on the basis of the above-mentioned topological analysis and the characteristics of transmembrane proteins (Figure 1A).

*Escherichia coli* PhoA has high activity in the periplasm but low activity in the cytoplasm, and therefore the fusions with this reporter protein are usually used to judge the orientation of TMHs of transmembrane proteins (Rothman et al., 1996; Meng L. et al., 2017). To determine the reliability of the above topological model, the orientation of N or C termini of UPF0118 was analyzed by using the PhoA assay. The fusions of UPF0118 with *E. coli* PhoA at the N or C termini were constructed, respectively, and then tested in the *phoA*-deficient *E. coli* mutant DH5α, using the empty vector or UPF0118 alone as the negative controls. On the LB medium plate containing 5-bromo-4-chloro-3-indolylphosphate as the substrate (Figure 1B, left panel), *E. coli* DH5α transformant expressing the construct UPF0118-C-PhoA showed significantly dark blue lawn whereas *E. coli* DH5α transformant expressing N-PhoA-UPF0118 showed weakly blue lawn. Also, the latter showed similar lawn color to those of *E. coli* DH5α transformants with the empty vector or expressing UPF0118 alone. This reveals that C terminus of UPF0118 is exactly located in the periplasm while N terminus of UPF0118 is located in the cytoplasm, as illustrated in Figure 1A. That was also confirmed by the alkaline phosphatase activity assay using the above-mentioned *E. coli* DH5α transformants (Figure 1B, right table). Based on the above results, the topological model of UPF0118 is reliable and suitable for the analysis of functionally important residues.

### Recognition of Five Conserved Motifs of UPF0118 by Sequence Alignment and Weblogo

In our previous study, we selected 27 representatives of *H. andaensis* UPF0118 homologs to analyze the phylogenetic relationship between UPF0118 and its homologs and known Na^+^/H^+^ antiporters. Unexpectedly, we found that UPF0118 shares five fully conserved motifs with nine phylogenetically closest homologs at a wide range of 58–82% identities (Dong et al., 2017). This suggests that these five motifs may be vital for the function of UPF0118. Therefore, we aligned UPF0118 and 27 representatives of its phylogenetically related homologs to further explore the conservation of residues located in these five motifs within a wider range of 30–82% identities. As expected, five highly conserved motif candidates were recognized as follows: (i) Motif A candidate with the consensus sequence of “Lv(i)SFLVYLIALFLFMLd(e)LPr(k)L”; (ii) Motif B candidate with the consensus sequence of “GFl(f/i)KAQFLVSi(l)IIF”; (iii) Motif C candidate with the consensus sequence of “DFi(v/l)PIi(l)GSI”; (iv) Motif D candidate with the consensus sequence of “IRRTVEPKVMGr(t/s)h(q/n)IGLS”; and (v) Motif E candidate with the consensus sequence of “k(r)EAGi(m/v)Ikw(m/f)NFK” (Supplementary Figure S1).

**FIGURE 2:**
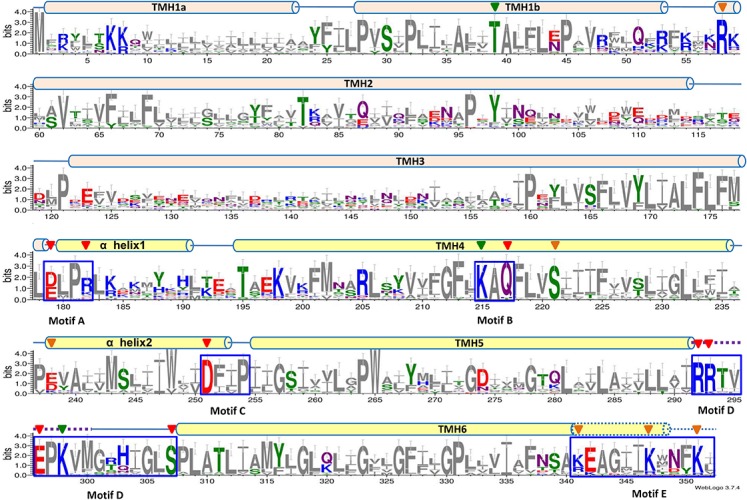
Weblogo of *Halobacillus andaensis* UPF0118 and its homologs. A weblogo was created by submitting the sequence alignment of *H. andaensis* UPF0118 with 27 representatives of its phylogenetically related homologs (Dong et al., 2017) to the website http://weblogo.threeplusone.com/create.cgi. Six putative transmembrane helices (TMH) and two α helices are marked with light pink (for TMHs1-3) and light yellow (for TMHs4-6 and α helices) filled cylinders above the alignment. Five highly conserved motifs (A to E) are highlighted within the blue dash-line border open rectangles. Membrane-buried residues in Loop5-6 are shown in the dotted line. Red filled downward arrows point to functionally important residues, green filled downward arrows point to pH response-related residues, and brown filled downward arrows point to functionally unrelated residues.

A weblogo was created to more clearly show the conservation of polar and charged residues located in these motifs (Figure 2). Notably, these five motif candidates are located within the range starting from α Helix 1 to TMH6. Within these five motif candidates, conserved polar or charged residues are E179 [relatively conserved between Glu (E) and Asp (D)] and R182 [relatively conserved between Arg (R) and Lys (K)] located in Motif A; K215, Q217, and S221 located in Motif B; D251 located in Motif C; R292, R293, E296, K298, and S307 located in Motif D; and K341 (similar relative conservation to R182), R347, and R351 located in Motif E (Figure 2). Outside the above five motif candidates, there are also some conserved polar or charged residues, such as T195, K198, R205, and E238 (similar relative conservation to E179) (Figure 2). However, there are almost no conserved consensuses within the former TMHs (TMHs1-3), except for one relatively conserved consensus sequence mainly located in TMH1b (Figure 2). Conserved polar or charged residues were quite rare within the former TMHs (TMHs1-3), other than T39, R51, T82, and etc. Therefore, we hypothesize that the above five motifs, especially their polar or charged residues, may play a vital role in the function of UPF0118 and even its homologs.

### UPF0118 Represents an Independent Na^+^/H^+^ Antiporter Group in AI-2E Family

**FIGURE 3:**
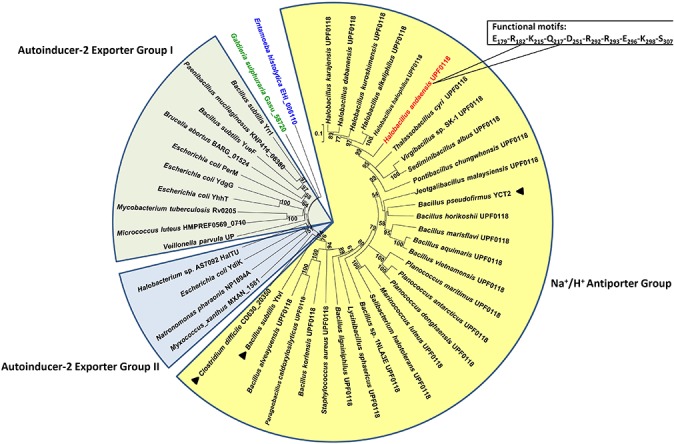
Phylogenetic relationship between UPF0118-type Na^+^/H^+^ antiporters and AI-2E family members. For the construction of phylogenetic tree, *H. andaensis* UPF0118 (TC#2.A.86.1.14) and 27 representatives of its phylogenetically related homologs (Dong et al., 2017) were selected, and also all the AI-2E family members (TC#2.A.86.1.1 to TC#2.A.86.2.3) were downloaded from the TCDB system. Three major groups are highlighted in yellow (for Na^+^/H^+^ antiporter group), light blue (for Autoinducer-2 exporter group I), and light green (for Autoinducer-2 exporter group II) filled fan-shaped sectors, respectively. Two separate clades are highlighted in blue for *Entamoeba histolytica* EHI_006110 and in green for *Galdieria sulphuraria* Gasu_58720. *H. andaensis* UPF00118 is highlighted in red, and three AI-2E family members clustered with UPF0118 are marked with black filled triangles. Bootstrap values ≥70% (based on 1000 replications) are shown at branch points. Bar, 0.1 substitutions per amino acid residue position.

In the TCDB system (Saier et al., 2016), UPF0118 has been categorized as the second characterized member into AI-2E family, since it contains a similar conserved domain of this family (formerly designated DUF20 due to its unknown function). In addition to UPF0118, *E. coli* YdgG is the first characterized AI-2E family member that has been reported to function as an AI-2E exporter (Herzberg et al., 2006). However, 17 AI-2E family members other than UPF0118 and *E. coli* YdgG have been yet functionally unknown (Saier et al., 2016). To determine whether the above-mentioned five conserved motifs exist in all the members of AI-2E family, UPF0118 was aligned with all other AI-2E family members collected in the TCDB system. Besides *Bacillus pseudofirmus* YCT2 sharing with 55% identity with UPF0118, all the other AI-2E family members exhibit a significant variation in polar or charged residues at the corresponding positions to five conserved motifs of UPF0118 (Supplementary Figure S2). This suggests that these five motifs may be signature motifs for the function of UPF0118 and its homologs as Na^+^/H^+^ antiporters. To test this hypothesis, we analyzed the phylogenetic relationship between UPF0118 and its homologs and other AI-2E family members by constructing a neighbor-joining phylogenetic tree. As a result, all the selected proteins clustered into three major groups, with the exception of two proteins forming two separate clades (Figure 3). Importantly, UPF0118 and its homologs constituted an independent cluster with the bootstrap value of 72%, together with three AI-2E family members including *B. pseudofirmus* YCT2, *B. subtilis* YvtI (23% identity with UPF0118) and *Clostridium difficile* CD630_20350 (19% identity with UPF0118) (Figure 3). This cluster is designated as Na^+^/H^+^ Antiporter Group due to UPF0118 functioning as a Na^+^/H^+^ antiporter, which is significantly distant with other two major groups designated Autoinducer-2 Exporter Group I and II, and another two independent clades (Figure 3).

### Selection of Candidate Residues for Site-Directed Mutagenesis

In Na^+^/H^+^ antiporters, polar or charged residues have been established to play critical roles in cation translocation, protonation, pH response, conformational stability, electrogenesis and etc. (Morino et al., 2010; Jiang et al., 2013b; Uzdavinys et al., 2017; Xu et al., 2018, 2019; Shao et al., 2018; Patino-Ruiz et al., 2019). In order to determine the functional motifs of UPF0118 as a Na^+^/H^+^ antiporter, all the conserved polar and charged residues located in the above five motif candidates were selected for the analysis of site-directed mutagenesis. Also, T39 and R58 were selected as representatives of conserved residues located within TMHs1-3 whereas E238 was selected as a representative of conserved residues located outside the above five motif candidates within the range from α Helix 1 to TMH6. Moreover, to reflect the response of *upf0118* to salts or alkaline pH, the previous construct, pET22b-UPF0118 (Dong et al., 2017), was modified by substituting T7 promoter with the native promoter of *upf0118* by PCR via a pair of primers listed in Supplementary Table S1. The resulting new construct designated as pET22b-P-UPF0118 was re-sequenced to confirm the accuracy of PCR. This plasmid was also determined by growth tests for salt tolerance and alkaline pH resistance. As a result, pET22b-P-UPF0118 was found to be able to express UPF0118 normally as a Na^+^(Li^+^)/H^+^ antiporter, as the previous construct, pET22b-UPF0118 (Supplementary Figure S3). This new construct designated as pET22b-P-UPF0118 was therefore used for the template of all the variants of UPF0118.

### Functional Importance of E179 and R182 Located in Motif A

**FIGURE 4:**
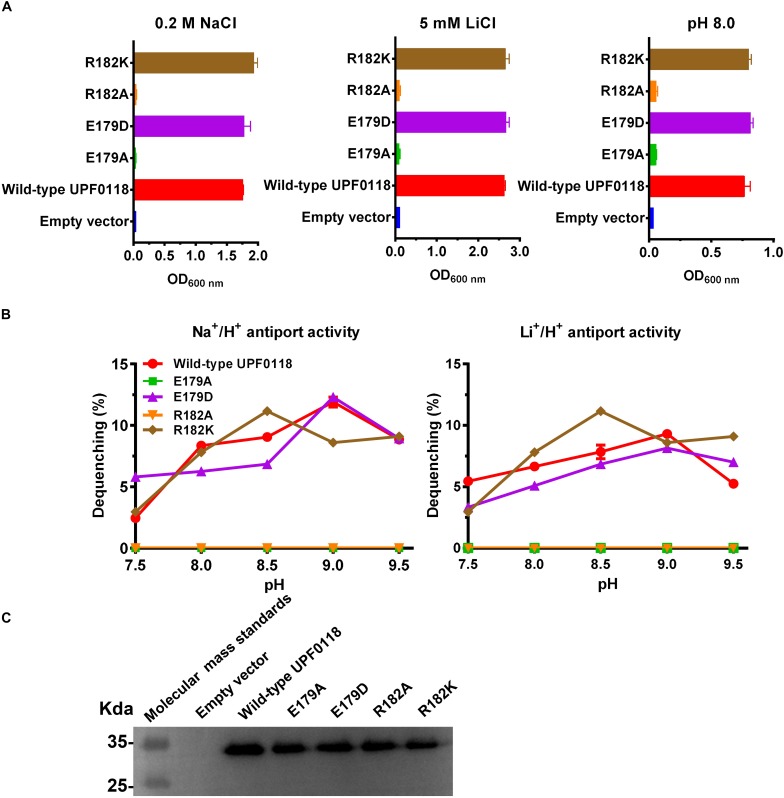
Functional analysis of E179 and R182 located in Motif A. Growth tests were carried out in the LBK media containing 0.2 M NaCl [**(A)**, left panel], 5 mM LiCl [**(A)**, middle panel] or at pH 8.0 plus 50 mM NaCl [**(A)**, right panel]. Each data point represents the average ± SD of three independent cultures. Na^+^/H^+^ [**(B)**, left panel] or Li^+^/H^+^ [**(B)**, right panel] antiport activities at the pH range of 7.5 to 9.5 were analyzed by using the everted membrane vesicles from *Escherichia coli* KNabc transformants expressing the tested variants, together with wild-type UPF0118 as the positive control. The expression levels **(C)** were also analyzed by using the everted membrane vesicles from the corresponding *E. coli* KNabc transformants.

*Escherichia coli* KNabc transformants expressing E179A or R182A completely lost the ability of growing in the presence of 0.2 M NaCl or 5 mM LiCl, or at pH 8.0 (Figure 4A), suggesting that side chains from E179 and R182 are vital for the function of UPF0118 as a Na^+^(Li^+^)/H^+^ antiporter. E179D or R182K restored the complementation with *E. coli* KNabc under the same stress conditions, as wild-type UPF0118 (Figure 4A), suggesting that the negative charge of side chain at No. 179 residue and the positive charge of side chain at No. 182 residue can satisfy the requirement of UPF0118 for Na^+^(Li^+^)/H^+^ antiport activity. The antiport activity analysis established the important roles of side chains from E179 and R182 in the Na^+^(Li^+^)/H^+^ antiport activity of UPF0118 (Figure 4B). Notably, substitution of R182 by lysine led to the shift of both Na^+^/H^+^ and Li^+^/H^+^ antiport activity profiles to acidic pH by 0.5, but retained similar *K*_0_._5_ values for Na^+^ and Li^+^ to those of wild-type UPF0118 (Table 1). This suggests that R182 may be involved in the response of antiport activity to pH. The above variants were determined by the western blot to be expressed in *E. coli* KNabc, as wild-type UPF0118 (Figure 4C).

**TABLE 1:** *K*_0__.5_ values of wild-type UPF0118 and its variants for Na^+^ and Li^+^.

	*K*_0__.5_ values^∗^ (mM)	
Variants/wild type	Na^+^	Li^+^
T39A	2.43 ± 0.36	1.51 ± 0.30
R58A	1.17 ± 0.23	1.30 ± 0.59
E179D	1.14 ± 0.20	1.45 ± 0.26
R182K	1.27 ± 0.16	1.58 ± 0.33
K215A	1.82 ± 0.44	1.41 ± 0.21
S221A	1.06 ± 0.17	2.06 ± 0.50
E238A	1.24 ± 0.28	1.92 ± 0.31
K298A	1.14 ± 0.30	1.40 ± 0.41
UPF0118	1.23 ± 0.28	1.51 ± 0.36

*^∗^*K*_0__.5_ values were calculated by plotting the antiport activity as the respective functions of cation concentrations, followed by a non-linear regression analysis.*

### Functional Analysis of K215, Q217, and S221 Located in Motif B

**FIGURE 5:**
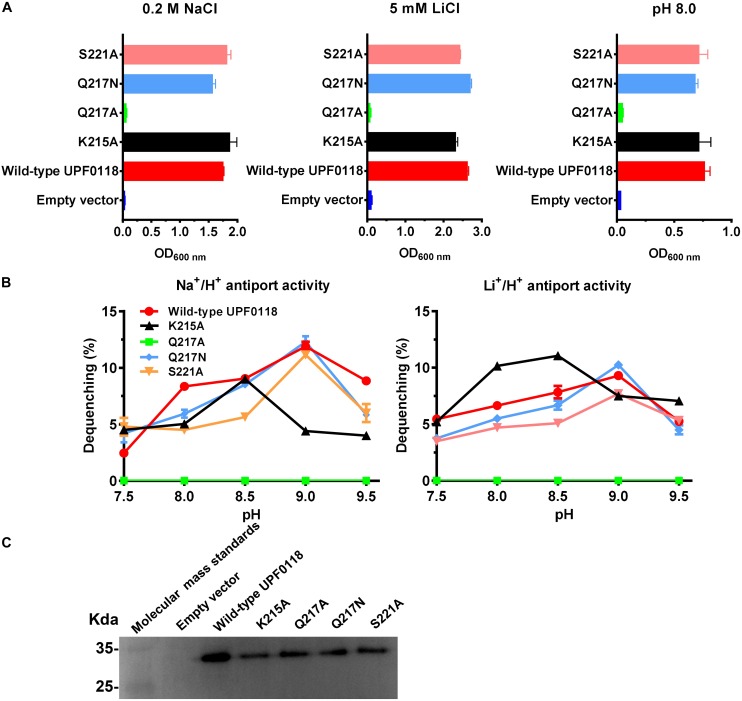
Functional analysis of the conserved residues located in Motif B. Growth tests were carried out in the LBK media containing 0.2 M NaCl [**(A)**, left panel], 5 mM LiCl [**(A)**, middle panel] or at pH 8.0 plus 50 mM NaCl [**(A)**, right panel]. Each data point represents the average ± SD of three independent cultures. Na^+^/H^+^ [**(B)**, left panel] or Li^+^/H^+^ [**(B)**, right panel] antiport activities at the pH range of 7.5 to 9.5 were analyzed by using the everted membrane vesicles from *Escherichia coli* KNabc transformants expressing the tested variants, together with wild-type UPF0118 as the positive control. The expression levels **(C)** were also analyzed by using the everted membrane vesicles from the corresponding *E. coli* KNabc transformants.

Under the tested stress conditions, Q217A could not, but Q217N could, offer the complementation ability with *E. coli* KNabc (Figure 5A). Also, the former completely lost both antiport activities whereas the latter restored similar activities to those of wild-type UPF0118 (Figure 5B). The combined results reveal that polar side chain from Q217 is vital for the Na^+^(Li^+^)/H^+^ antiport activity of UPF0118. K215A offered the same growth of *E. coli* KNabc as that of wild-type UPF0118 (Figure 5A). However, this variant exhibited significantly lower Na^+^/H^+^ antiport activity but showed similar Li^+^/H^+^ antiport activity, as compared with wild-type UPF0118 (Figure 5B). This variant possessed higher *K*_0_._5_ value for Na^+^ than that of wild-type UPF0118 while retained similar *K*_0_._5_ value for Li^+^ to that of the latter (Table 1). This indicates that K215 may be solely involved in the Na^+^ translocation. Interestingly, this variant also showed both activity profiles to acidic pH by 0.5, suggesting that K215 may be involved in the response of antiport activity to pH. S221A offered the same growth of *E. coli* KNabc (Figure 5A) and antiport activity (Figure 5B) as those of wild-type UPF0118, indicating that this residue should be unrelated to the function of UPF0118. The western blot also showed that the above variants were normally expressed in *E. coli* KNabc (Figure 5C).

### D251 Located in Motif C Is Indispensable for the Function of UPF0118

**FIGURE 6:**
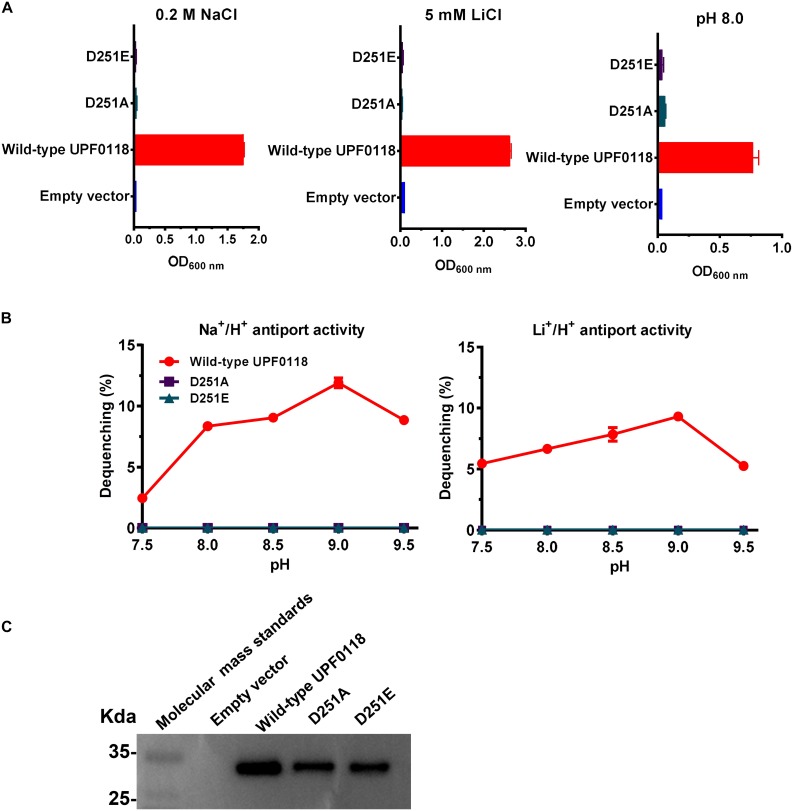
Functional analysis of D251 located in Motif C. Growth tests were carried out in the LBK media containing 0.2 M NaCl [**(A)**, left panel], 5 mM LiCl [**(A)**, middle panel] or at pH 8.0 plus 50 mM NaCl [**(A)**, right panel]. Each data point represents the average ± SD of three independent cultures. Na^+^/H^+^ [**(B)**, left panel] or Li^+^/H^+^ [**(B)**, right panel] antiport activities at the pH range of 7.5 to 9.5 were analyzed by using the everted membrane vesicles from *Escherichia coli* KNabc transformants expressing the tested variants, together with wild-type UPF0118 as the positive control. The expression levels **(C)** were also analyzed by using the everted membrane vesicles from the corresponding *E. coli* KNabc transformants.

D251A or D251E failed to complement with *E. coli* KNabc under the tested stress conditions, when wild-type UPF0118 offered normal complementation with *E. coli* KNabc (Figure 6A). Substitution of D251 by alanine abolished both Na^+^/H^+^ and Li^+^/H^+^ antiport activities of UPF0118, and substitution of this residue by glutamic acid could not recover either antiport activity (Figure 6B). The western blot ruled out the possibility that the loss of antiport activities is due to non-expression of either variant (Figure 6C). The above results reveal that D251 should act as a determining role in the Na^+^(Li^+^)/H^+^ antiport activity of UPF0118. More importantly, both negative charge and the length of side chain from this residue should be indispensable for the function of UPF0118.

### Functional Analysis of Conserved Polar and Charged Residues Located in Motif D

**FIGURE 7:**
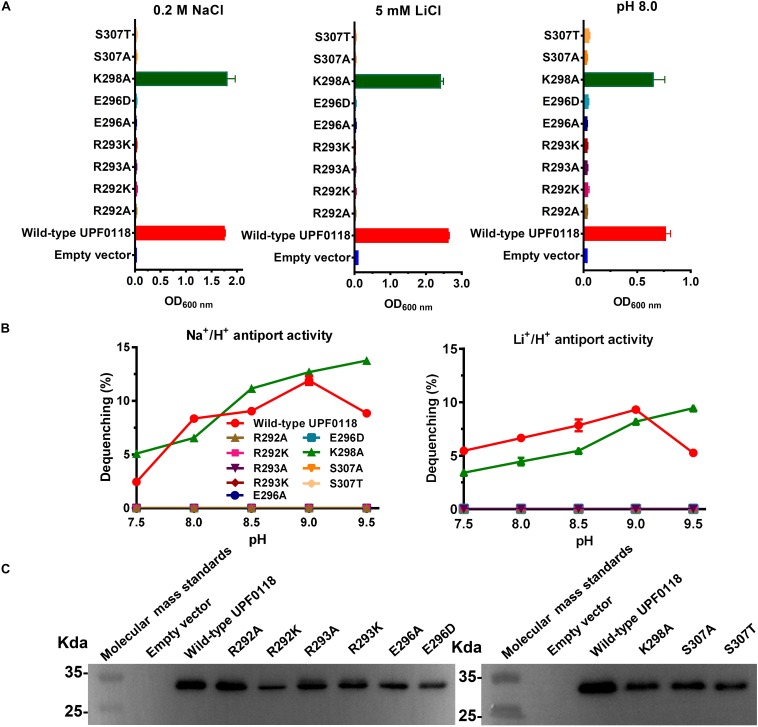
Functional analysis of the conserved residues located in Motif D. Growth tests were carried out in the LBK media containing 0.2 M NaCl [**(A)**, left panel], 5 mM LiCl [**(A)**, middle panel] or at pH 8.0 plus 50 mM NaCl [**(A)**, right panel]. Each data point represents the average ± SD of three independent cultures. Na^+^/H^+^ [**(B)**, left panel] or Li^+^/H^+^ [**(B)**, right panel] antiport activities at the pH range of 7.5 to 9.5 were analyzed by using the everted membrane vesicles from *Escherichia coli* KNabc transformants expressing the tested variants, together with wild-type UPF0118 as the positive control. The expression levels **(C)** were also analyzed by using the everted membrane vesicles from the corresponding *E. coli* KNabc transformants.

Substitution of R292 or R293 by alanine resulted in the complete loss of complementation ability of UPF0118 with *E. coli* KNabc and its Na^+^(Li^+^)/H^+^ antiport activity (Figures 7A,B). Also, substitution of either residue by lysine was unable to restore the complementation ability of UPF0118 with *E. coli* KNabc or its Na^+^(Li^+^)/H^+^ antiport activity (Figures 7A,B). This indicates that substitution of both residues by positively charged lysine can’t satisfy the requirement of UPF0118 to function as a Na^+^(Li^+^)/H^+^ antiporter. Namely, in addition to positive charge, nitrogen atom or amino group in guanidyl groups of side chains may also play a vital role in the Na^+^(Li^+^)/H^+^ antiport activity of UPF0118. Similarly, growth tests and antiport activity assays for E296A and E296D (Figures 7A,B) reveal that both negative charge and the length of this residue are vital for the normal function of UPF0118. K298A offered the same growth of *E. coli* KNabc as that of wild-type UPF0118 under the tested stress conditions (Figure 7A). However, both antiport activity profiles for K298A were shifted to alkaline pH by 0.5 (Figure 7B) while this variant retained similar *K*_0_._5_ values for Na^+^ and Li^+^ to those of wild-type UPF0118 (Table 1). These results reveal that this residue may be involved in the response of antiport activity to pH. S307A or S307T failed to complement with *E. coli* KNabc under the tested stress conditions (Figure 7A) and completely lost Na^+^(Li^+^)/H^+^ antiport activity (Figure 7B). This indicates that both hydroxyl group and the length of this polar residue are indispensable for the Na^+^(Li^+^)/H^+^ antiport activity of UPF0118. The western blot established normal expression of the above variants in *E. coli* KNabc (Figure 7C).

### Functional Analysis of C Terminus and Three Basic Residues Located in Motif E

**FIGURE 8:**
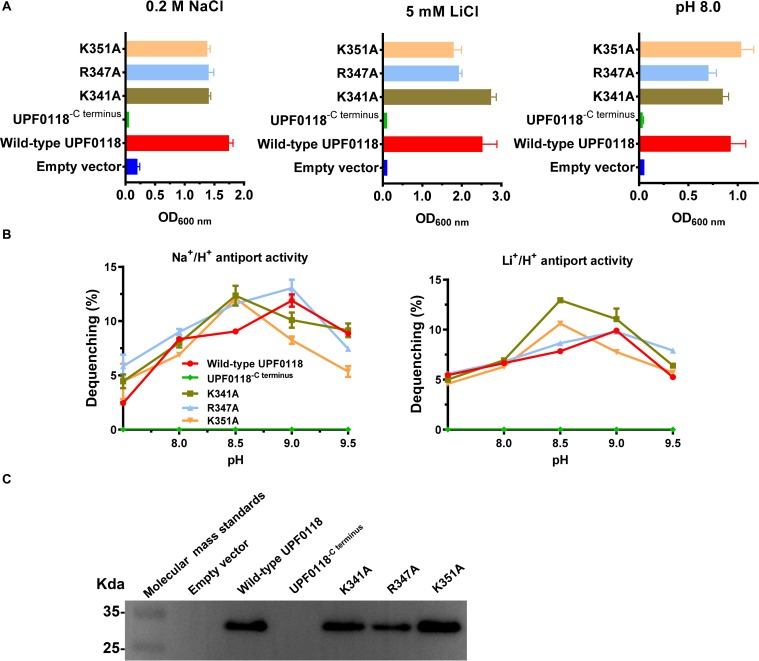
Functional analysis of C terminus and three alkaline residues located in Motif E. Growth tests were carried out in the LBK media containing 0.2 M NaCl [**(A)**, left panel], 5 mM LiCl [**(A)**, middle panel] or at pH 8.0 plus 50 mM NaCl [**(A)**, right panel]. Each data point represents the average ± SD of three independent cultures. Na^+^/H^+^ [**(B)**, left panel] or Li^+^/H^+^ [**(B)**, right panel] antiport activities at the pH range of 7.5 to 9.5 were analyzed by using the everted membrane vesicles from *Escherichia coli* KNabc transformants expressing the tested variants, together with wild-type UPF0118 as the positive control. The expression levels **(C)** were also analyzed by using the everted membrane vesicles from the corresponding *E. coli* KNabc transformants.

A C terminus-truncated variant, UPF0118^−*C*^^*terminus*^, failed to complement with *E. coli* KNabc, and no antiport activity was detected from everted membrane vesicles of *E. coli* KNabc expressing this variant. However, substitution of K341, R347 or K351 by alanine had no effect on their complementation ability of UPF0118 with *E. coli* KNabc (Figure 8A) or its antiport activities (Figure 8B). The western blot showed that UPF0118^−*C*^^*terminus*^ could not be expressed in *E. coli* KNabc while each of K341A, R347A or K351A was expressed as wild-type UPF0118 (Figure 8C). UPF0118^−*C*^^*terminus*^ was constructed by deleting the residues from K341 to the end of C terminus. Since this truncated variant was unable to be successfully expressed and substitution of K341, R347 or K351 by alanine did not affect the Na^+^(Li^+^)/H^+^ antiport activity of UPF0118, the truncated region in Motif E should determine the normal expression and even accurate localization of UPF0118 into the cytoplasmic membranes. Most of the multiple web-based softwares predicted that the last TMH of UPF0118 ends at A340 or the former residues (Supplementary Table S2) whereas PredictProtein predicted that TMH6 ends at W348 (Figure 1A and Supplementary Table S3). Therefore, the above results support the accuracy of newly predicted topological model of UPF0118 by PredictProtein. This also implies that Motif E may not be a signature motif to determine the function of UPF0118 as a Na^+^(Li^+^)/H^+^ antiporter.

### Functional Analysis of T39, R58, and E238 Located Outside the Five Conserved Motifs

Substitution of T39, R58, or E238 by alanine had no effect on the complementation ability of UPF0118 with *E. coli* KNabc (Supplementary Figure S4A). Also, each of R58A and E238A retained similar Na^+^(Li^+^)/H^+^ antiport activity to that of wild-type UPF0118 (Supplementary Figure S4B). However, T39A exhibited significantly lower Na^+^/H^+^ antiport activity than that of wild-type UPF0118 but similar Li^+^/H^+^ antiport activity to that of the latter (Supplementary Figure S4B). This suggests that T39 may be involved in Na^+^ translocation. Also, both antiport activity profiles of T39A were shifted to acidic pH by 0.5 (Supplementary Figure S4B). This suggests that this residue may be involved in the response of antiport activity to pH. The western blot showed the normal expression of the above variants in *E. coli* KNabc (Supplementary Figure S4C). Since mutation in the above three residues did not affect the complementation ability of UPF0118 with *E. coli* KNabc (Supplementary Figure S4A), T39, R58 and E238 should not be key functional residues for UPF0118 as a Na^+^(Li^+^)/H^+^ antiporter. It seems that polar or charged residues outside five conserved motifs may not play a determining role in the function of UPF0118 as a Na^+^(Li^+^)/H^+^ antiporter. This indirectly supports the possibility that conserved motifs can be used for signature functional motifs.

## Discussion

In this study, we presented that a previously characterized Na^+^/H^+^ antiporter UPF0118, together with its homologs, should represent an independent group of AI-2E family. This group was proposed to designate as Na^+^/H^+^ Antiporter Group, which exhibits a distant phylogenetic relationship with the majority of AI-2E members collected in the TCDB system (Saier et al., 2016). Notably, Na^+^/H^+^ Antiporter Group shows five highly conserved motifs, which are not detected in the majority of AI-2E family members collected in the TCDB system. Functional analysis established that polar or charged residues located in Motifs A to D play a vital role in the Na^+^(Li^+^)/H^+^ antiport activity or pH response of UPF0118. However, three basic residues located in Motif E are not involved in the function of UPF0118, although the truncation of C terminus resulted in the non-expression of this transporter. Therefore, we propose that E_179_-R_182_-K_215_-Q_217_-D_251_-R_292_-R_293_-E_296_-K_298_-S_30__7_ located within five functional motifs can be used for signature functional motifs to recognize whether AI-2E family members function as Na^+^(Li^+^)/H^+^ antiporters.

UPF0118 family was formerly a functionally uncharacterized protein family with No. 0118, members of which contain a conserved domain of unknown function, DUF20 (Marchler-Bauer et al., 2015). This family has been re-designated as AI-2E family since *E. coli* YdgG was reported to function as an AI-2 exporter (Herzberg et al., 2006). In addition to *E. coli* YdgG, UPF0118 is the second characterized AI-2E family member (Saier et al., 2016). Moreover, *B. subtilis* YtvI was reported to be related to the spore formation of host strain, despite being functionally uncharacterized (Eichenberger et al., 2003). So far, 19 AI-2E family members collected in the TCDB system share quite low identities (Saier et al., 2016). Our results showed that UPF0118 has low identities from 13% to 23% with these members, with one exception of *B. pseudofirmus* YCT2 (Supplementary Figure S2). Phylogenetic analysis suggests that UPF0118 and its homologs may represent an independent group of AI-2E family (Figure 3). Due to UPF0118 functioning as a Na^+^/H^+^ antiporter, this group is therefore designated as Na^+^/H^+^ Antiporter Group. Also, *B. pseudofirmus* YCT2, *B. subtilis* YvtI and *C. difficile* CD630_20350 may exhibit similar protein functions to that of UPF0118 as a Na^+^/H^+^ antiporter, which are different from *E. coli* YdgG and other members functioning as AI-2Es.

UPF0118 and its homologs showed five highly conserved motifs, Motifs A to E (Figure 2 and Supplementary Figure S1). Notably, there is a significant variation in polar charged residues of Motifs A to E between UPF0118 and AI-2E family members excluding *B. pseudofirmus* YCT2 (Supplementary Figure S2). This suggests that these motifs may be used to differentiate the members of Na^+^/H^+^ Antiporter Group from all the AI-2E family members. Our results showed that E179 and R182 in Motif A could be substituted with Asp and Lys, respectively, for the normal function of UPF0118 as a Na^+^(Li^+^)/H^+^ antiporter (Figure 4). Similarly, Q217 in Motif B can also be substituted with asparagine (Figure 5). In contrast, each of D251 in Motif C, and R292, R293, E296, and S307 in Motif D can’t be substituted with the corresponding negatively charged residue or positively charged residue (Figures 6, 7). However, three basic residues located in Motif E are not involved in the function of UPF0118, although the truncation of C terminus resulted in the non-expression of this transporter. Therefore, we propose that E_179_-R_182_-K_215_-Q_217_-D_251_-R_292_-R_293_-E_296_-K_298_-S_30__7_ of UPF0118 located in Motifs A to D can be used for signature functional motifs to recognize whether AI-2E family members function as Na^+^(Li^+^)/H^+^ antiporters. Moreover, K215 in Motif B or K298 in Motif D can’t determine the Na^+^(Li^+^)/H^+^ antiport activity of UPF0118, though either one plays a vital role in the response of antiport activity to pH (Figures 5, 7). Also, No. 179 residue in Motif A can be switched between Asp and Glu whereas No. 182 residue in Motif A may be switched between Arg and Lys. Similarly, No. 217 residue in Motif B may be switched between Gln and Asn. These results imply that one AI-2E family member may function as a Na^+^/H^+^ antiporter if it possesses the same conserved motifs with E_179_(D)-R_182_(K)-K_215_(A)-Q_217_(N)-D_251_-R_292_-R_293_-E_296_-K_298_(A)-S_30__7_.

Interestingly, three basic residues of UPF0118, R182, K215, and K298, are involved in the response of Na^+^(Li^+^)/H^+^ antiport activity to pH (Figures 4, 5, 7). That was rarely reported in Na^+^(Li^+^)/H^+^ antiporters, except for K305 in *Thermus thermophilus* NapA (Uzdavinys et al., 2017). This suggests that UPF0118 may exhibit a different pH response mechanism of Na^+^(Li^+^)/H^+^ antiport activity. Also, there are two α helices and six TMHs in the topological structure of UPF0118 (Figure 1A), suggesting that this transporter may have a different structural fold from NhaA fold representing a classic 3D structure of Na^+^/H^+^ antiporters (Hunte et al., 2005). Notably, E179 and R182 in α helix I and D251 in α helix II play a vital role in the function of UPF0118 (Figures 4, 6). Moreover, a buried loop designated as Loop5-6 contains five functionally important or pH response-related residues, R292, R293, E296, K298, and S307 (Figure 1A). This implies that UPF0118 may employ a novel molecular mechanism of Na^+^, Li^+^ transporting and pH response. This may be a different molecular mechanism from that of characterized Na^+^/H^+^ antiporters distributed in ten major families or superfamilies in the TCDB system, since AI-2E family is a significantly different newly characterized protein family (Herzberg et al., 2006; Saier et al., 2016; Dong et al., 2017). In the future study, we plan to purify and over-express UPF0118, and analyze 3D crystal structure of UPF0118 to discover its molecular mechanism of Na^+^, Li^+^ transporting and pH response.

Taken together, the results presented in this study provide a strong evidence for UPF0118 and its homologs to represent an independent group of AI-2E family, Na^+^/H^+^ Antiporter Group. More importantly, we propose that E_179_-R_182_-K_215_-Q_217_-D_251_-R_292_-R_293_-E_296_-K_298_-S_30__7_ of UPF0118 can be used for signature functional motifs to recognize AI-2E family members functioning as Na^+^(Li^+^)/H^+^ antiporters. Current findings not only trigger the understanding of molecular mechanism of Na^+^, Li^+^ transporting and pH response of UPF0118, but also clarify the knowledge of AI-2E family including a large number of uncharacterized members.

## Data Availability Statement

Publicly available datasets were analyzed in this study. This data can be found in GenBank under accession no. KY231907.

## Author Contributions

JJ, LW, and QZ designed the experiments. LW and QZ constructed the subclone and variants. LW, QZ, MY, and HC performed the topological analysis of UPF0118. LW, QZ, MY, YW, SG, RZ, YS, XL, HC, LS, and LM carried out the growth tests, activity assays, or western blots. JJ wrote the manuscript. All authors checked and approved the final version of this manuscript.

## Conflict of Interest

The authors declare that the research was conducted in the absence of any commercial or financial relationships that could be construed as a potential conflict of interest.
