# Supplementary material for: Polar or Charged Residues Located in Four Highly Conserved Motifs Play a Vital Role in the Function or pH Response of a UPF0118 Family Na+(Li+)/H+ Antiporter
Source: Front Microbiol. 2020 May 7;11:841. doi: 10.3389/fmicb.2020.00841 (PMC7221264; doi:10.3389/fmicb.2020.00841)
Supplement: Supplementary file 1 [file Data_Sheet_1.docx]

Supplementary Material

Polar or charged residues located in four highly conserved motifs play a vital role in the function or pH response of a UPF0118 family Na^+^(Li^+^)/H^+^ antiporter.

Lidan Wang^#^, Qiao Zou^#^, Mingxue Yan, Yuting Wang, Sijia Guo, Rui Zhang, Yang Song, Xiaofang Li, Huiwen Chen, Li Shao, Lin Meng, Juquan Jiang*

^Department of Microbiology and Biotechnology, College of Biological Sciences, Northeast Agricultural University, No. 600 Changjiang Road, Xiangfang District, Harbin, China, 150030^

*** Correspondence:**Juquan Jiang
jjqdainty@163.com

**^#^ Authors equally contribute to this work.**

**Supplemental Table 1. Strains, plasmids and primers used in this study.**

| **Strains, plasmids and primers** | **Relevant phenotype，genotype or primer sequences (5' to 3')** | **Source** |
| --- | --- | --- |
| **Strains** |  |  |
| *Escherichia coli* KNabc | *E. coli* mutant, lacking three major Na^+^/H^+^ antiporters (NhaA, NhaB and ChaA) | Donated by Professor Terry A. Krulwich (Nozaki et al., 1996) |
| *Escherichia coli* DH5α | F^–^ *endA1* *glnV44* *thi-1* *recA1* *relA1* *phoA gyrA96* *deoR* *nupG* *purB20* φ80d*lacZ*ΔM15 Δ(*lacZYA-argF*)U169, hsdR17(*r_K_*^–^*m_K_*^+^), λ^–^ | Promega (Beijing) Biotech Co., Ltd. |
| Plasmids |  |  |
| pEasy T3 | Cloning vector | TransGen Biotech. China |
| pET22b | Over-expression vector | Novagen Co. Ltd |
| pET22b- UPF0118 | pET-22b carrying *Halobacillus andaensis* UPF0118 fused with an N-terminal His_6_ tag | Constructed in our previous study (Dong et al., 2017) |
| pET22b-P-UPF0118 | pET22b carrying *Halobacillus andaensis* UPF0118 fused with an N-terminal His_6_ tag, with T7 promoter substituted by the native promoter (P) of *upf0118* | This study |
| pEasy T3-P-PhoA-UPF0118 | pEasy T3 with the construct P-N-PhoA-UPF0118 | This study |
| pEasy T3-P-UPF0118-PhoA | pEasy T3 with the construct Pro-UPF0118-C-PhoAang | This study |
| **Primers** |  |  |
| Promoter_F | AGATCTTGGTATTGAGGGACTTGTTG (*Bgl*II site, underlined) | This study |
| Promoter_R | TCTAGATGTAAACTGAATCCTCCTTC (*Xba*l site, underlined) | This study |
| UPF0118_F | GGATCCGATGTTCCGCTACCTTTCG (*Bam*HI site, underlined) | This study |
| UPF0118_R | CTCGAGTTATAATTTAAAGTTCCAG (*Xho*I site, underlined) | This study |
| P-N-PhoA _F | TGGTATTGAGGGACTTGTTGTTGAT | This study |
| P-N-PhoA _OR | GGCATTTCTGGTGTCCGCATGCTGCTGCCCATGGTATATCTCCTT (overlapping PhoA starting from No. 39 residue, underlined) | This study |
| P-N-PhoA _OF | GATATACCATGGGCAGCAGCATGCGGACACCAGAAATGCCTGTTC (overlapping PhoA starting from No. 39 residue, underlined) | This study |
| P-N-PhoA _R | TTTCAGCCCCAGAGCGGCTTTCATG | This study |
| N-PhoA-UPF0118_OR | CTGTGATGATGATGATGATGTTTCAGCCCCAGAGCGGCTTTCATG (overlapping PhoA at the C terminus without TAA, underlined) | This study |
| N-PhoA-UPF0118_OF | AAGCCGCTCTGGGGCTGAAACATCATCATCATCATCACAGCAGCG (overlapping PhoA at the C terminus without TAA, underlined) | This study |
| N-PhoA-UPF0118_R | TTATAATTTA AAGTTCCAGCGGATA | This study |
| UPF0118-C-PhoA_OR | GGCATTTCTGGTGTCCGCATTAATTTAAAGTTCCAGCGGATAATA (overlapping PhoA starting from No. 39 residue, underlined) | This study |
| UPF0118-C-PhoA_OF | TCCGCTGGAACTTTAAATTAATGCGGACACCAGAAATGCCTGTTC (overlapping PhoA starting from No. 39 residue, underlined) | This study |
| C-PhoA_R | TTATTTCAGCCCCAGAGCGGCTTTC | This study |
| T39A_F | ATCATCGCTTTAATTGCAGCCCTCTTT (mutated bases, underlined) | This study |
| T39A_R | CAATTAAAGCGATGATTAAGGGAACT (mutated bases, underlined) | This study |
| R58A_F | AGATTTAGGCTCAATGCAAAAATGGC (mutated bases, underlined) | This study |
| R58A_R | GCATTGAGCCTAAATCTAAACTGCATC (mutated bases, underlined) | This study |
| E179A_F | TTCTCTTTATGCTAGCATTGCCTCGC (mutated bases, underlined) | This study |
| E179A_R | GCTAGCATAAAGAGAAATAGCGCAA (mutated bases, underlined) | This study |
| E179D_F | TCTCTTTATGCTAGACTTG CCTCGCT (mutated bases, underlined) | This study |
| E179D_R | GTCTAGCATAAAGAGAAATAG CGCA (mutated bases, underlined) | This study |
| R182A_F | ATGCTAGAATTGCCTGCCTTAAAGGAT (mutated bases, underlined) | This study |
| R182A_R | GCAGGCAATTCTAGCATAAAGAGAAA (mutated bases, underlined) | This study |
| R182K_F | ATGCTAGAATTGCCTAAGTTAAAGGAT (mutated bases, underlined) | This study |
| R182K_R | CTTAGGCAATTCTAGCATAAAGAGAA (mutated bases, underlined) | This study |
| K215A_F | GTGTTTGGTTTCTTAGCAGCTCAATTC (mutated bases, underlined) | This study |
| K215A_R | GCTAAGAAACCAAACACAACGTAA (mutated bases, underlined) | This study |
| Q217A_F | GGTTTCTTAAAAGCTGCATTCCTTGTT (mutated bases, underlined) | This study |
| Q217A_R | GCAGCTTTTAAGAAACCAAACACAAC (mutated bases, underlined) | This study |
| Q217N_F | TTAAAAGCTAACTTCCTTGTTAGTATC (mutated site, underlined) | This study |
| Q217N_R | GTTAGCTTTTAAGAAACCAAACACAAC(mutated site, underlined) | This study |
| S221A_F | GCTCAATTCCTTGTTGCTATCGTTATC (mutated bases, underlined) | This study |
| S221A_R | GCAACAAGGAATTGAGCTTTTAAGAA (mutated bases, underlined) | This study |
| E238A_F  | TTTGGATTACTCCAGCAGTTGCAATC (mutated base, underlined) | This study |
| E238A_R | GCTGGAGTAATCCAAAACAACCCGA (mutated base, underlined) | This study |
| D251A_F | TTATTTGGATTGTAGCCTTCGTCCCA (mutated bases, underlined) | This study |
| D251A_R | GCTACAATCCAAATAATAAGAGACA (mutated bases, underlined) | This study |
| D251E_F | TTATTTGGATTGTAGAATTCGTCCCAA (mutated bases, underlined) | This study |
| D251E_R | TTCTACAATCCAAATAATAAGAGACA (mutated bases, underlined) | This study |
| R292A_F | ATCCTGCTGGCCATAGCACGAACCGTA (mutated bases, underlined) | This study |
| R292A_R | GCTATGGCCAGCAGGATAATGGCCAA (mutated bases, underlined) | This study |
| R292K_F | CTGGCCATAAAGCGAACCGTAGAACCT (mutated bases, underlined) | This study |
| R292K_R | CTTTATGGCCAGCAGGATAATGGCCAA (mutated bases, underlined) | This study |
| R293A_F | CTGCTGGCCATAAGAGCAACCGTAGA (mutated bases, underlined) | This study |
| R293A_R | GCTCTTATGGCCAGCAGGATAATGGC (mutated bases, underlined) | This study |
| R293K_F | GCCATAAGAAAGACCGTAGAACCTAAG (mutated bases, underlined) | This study |
| R293K_R | CTTTCTTATGGCCAGCAGGATAATGGC (mutated bases, underlined) | This study |
| E296A_F | TAAGACGAACCGTAGCACCTAAGGTG (mutated base, underlined) | This study |
| E296A_R | GCTACGGTTCGTCTTATGGCCAGCA (mutated base, underlined) | This study |
| E296D_F | CGAACCGTAGACCCTAAGGTGATGGGG (mutated base, underlined) | This study |
| E296D_R | GTCTACGGTTCGTCTTATGGCCAGCAG (mutated base, underlined) | This study |
| K298A_F | CGAACCGTAGAACCTGCGGTGATGGG (mutated bases, underlined) | This study |
| K298A_R | GCAGGTTCTACGGTTCGTCTTATGGC (mutated bases, underlined) | This study |
| S307A_F | CGCCATATCGGACTCGCTCCACTAGC (mutated base, underlined) | This study |
| S307A_R | CGAGTCCGATATGGCGCCCCATCAC (mutated base, underlined) | This study |
| S307T_F | ATCGGACTCACACCACTAGCTACCCTT (mutated bases, underlined) | This study |
| S307T_R | TGTGAGTCCGATATGGCGCCCCATCAC (mutated bases, underlined) | This study |
| Minus C terminus_F | the same as Pro_F | This study |
| Minus C terminus_R | CTCGAGTTATGCAGAATTAAAGGCGATG (*Xho*I site, underlined) | This study |
| K341A_F | AATTCTGCAGCAGAAGCAGGTATTATC (mutated bases, underlined) | This study |
| K341A_R | TGCTGCAGAATTAAAGGCGATGACAAG (mutated bases, underlined) | This study |
| R347A_F | GGTATTATCGCCTGGAACTTTAAATTA (mutated bases, underlined) | This study |
| R347A_R | GGCGATAATACCTGCTTCTTTTGCAGA (mutated bases, underlined) | This study |
| K351A_F | TGGAACTTTGCATTATAACTCGAGCAC (mutated bases, underlined) | This study |
| K351A_R | TGCAAAGTTCCAGCGGATAATACCTGC (mutated bases, underlined) | This study |

**Supplemental Table 2. Topological analysis of *Halobacillus andaensis* UPF0118 using the multiple web-based softwares as follows.**

|  | **HMMTOP** | **TMHMM** | **TMPred** | | **PRED-TMR** | **SOSUI** | **Phyre 2** |
| --- | --- | --- | --- | --- | --- | --- | --- |
| Orientation of N, C termini |  | N_out_-C_in_ | N_in_-C_out_ | N_out_-C_in_ |  |  | N_in_-C_in_ |
| TMH1 | 11 - 36 | 10 - 43 | 10 - 29 | 10 - 27 | 10 - 27 | 17 - 39 | 10 - 26 |
| TMH2 | 61 - 86 | 64 - 86 | 60 - 79 | 60 - 80 | 29 - 47 | 60 - 82 | 30 - 47 |
| TMH3 | 152 - 177 | 159 - 178 | 163 - 181 | 162 - 179 | 59 - 77 | 154 - 172 | 60 - 89 |
| TMH4 | 206 - 231 | 208 - 230 | 218 - 234 | 218 - 234 | 162 - 178 | 221 - 243 | 150 - 177 |
| TMH5 | 239 - 264 | 240 - 262 | 245 - 267 | 246 - 273 | 218 - 235 | 270 - 291 | 212 - 235 |
| TMH6 | 270 - 292 | 269 - 291 | 265 - 291 | 269 - 291 | 239 - 261 | 309 - 331 | 241 - 271 |
| TMH7 | 312 - 337 | 315 - 337 | 312 - 337 | 317 - 337 | 269 - 291 |  | 278 - 293 |
| TMH8 |  |  |  |  | 321 - 337 |  | 310 - 340 |

**Supplemental Table 3.** **Topological analysis of *Halobacillus andaensis* UPF0118 using PredictProtein.**

|  | | **PredictProtein** | |
| --- | --- | --- | --- |
| **No.** | **Helix region** | **TMH region** | **Buried region** |
| 1 | 2 - 53 | 15 - 39 | 4 - 46 |
| 2 | 58 - 114 | 60 - 85 | 57 - 84 |
| 3 | 122 - 178 | 148 - 178 | 150 - 179 |
| 4 | 180 - 190 |  |  |
| 5 | 195 - 235 | 214 - 235 | 208 - 235 |
| 6 | 238 - 252 |  | 238 - 272 |
| 7 | 255 - 291 | 266 - 288 | 274 - 290 |
| 8 |  |  | 292 - 300 |
| 9 | 308 - 348 | 308 - 326/328 - 346 | 305 - 347 |


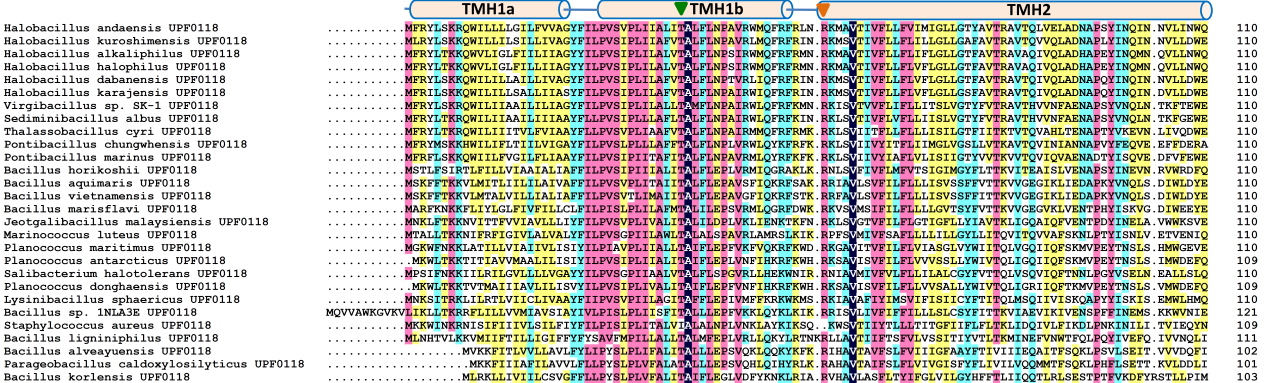

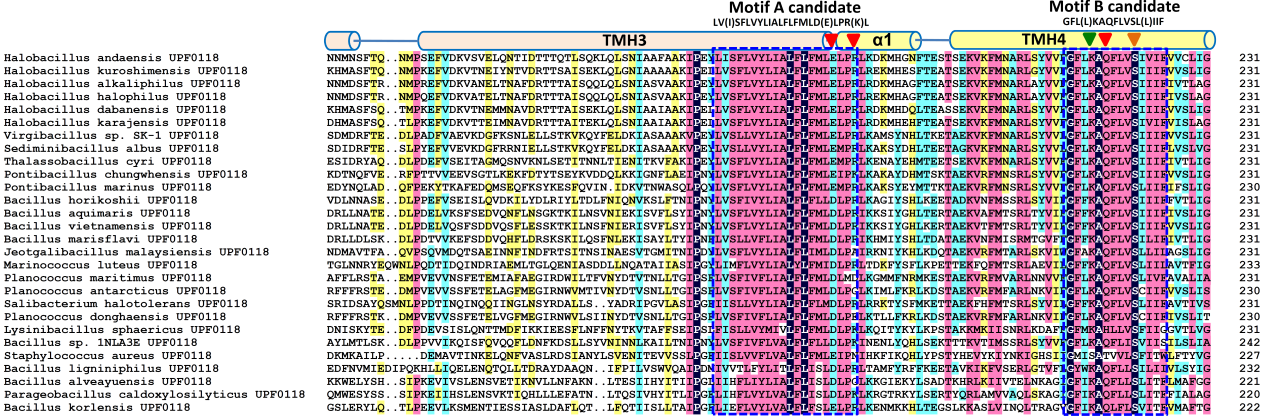

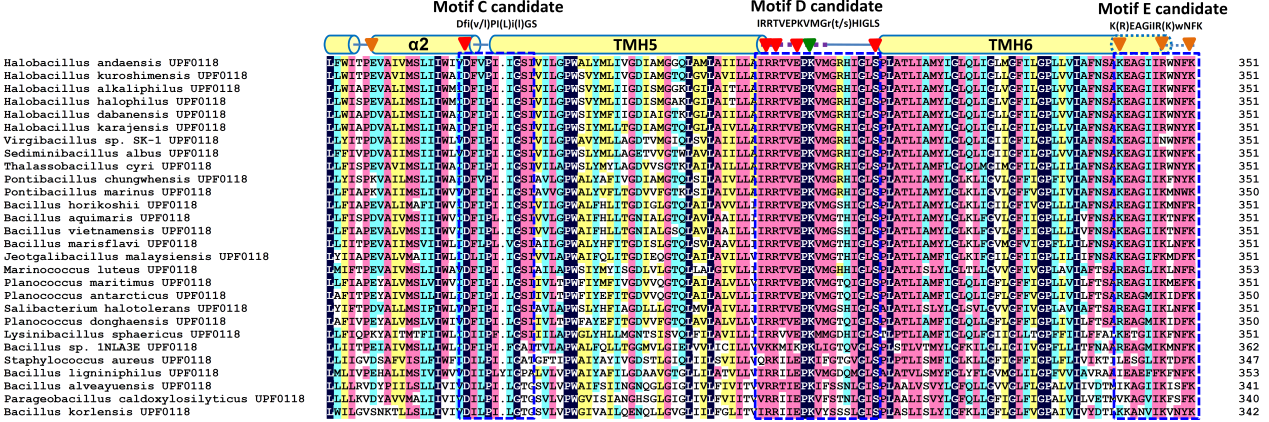


**Supplemental Figure 1. Alignment of UPF0118 with its phylogenetically-related representative homologs.**

UPF0118 was aligned with 27 representatives of its phylogenetically-related homologs clustered with the bootstrap value of 99% in our previous study (Dong et al., 2017). Shading homology corresponds to 100% (black), ≥ 75% (pink), ≥ 50% (cyan), ≥ 33% (yellow) and < 33% (white) amino acid identity, respectively. Six putative transmembrane helices (TMH) and two α helices are marked with the light pink (for TMH1-3) and light yellow (for TMH4-6 and α helices) filled cylinders above the alignment. Five highly conserved motif (A to E) candidates are highlighted within blue dash-line border open rectangles. The corresponding consensus sequences of the motifs are shown as follows: capital letters stand for residues with the frequency of occurrence at or above 75%; lowercase letters stand for residues with the frequency of occurrence at or above 50%, together with the substituted residues in the parentheses. Red filled downward arrows point to the functionally important residues, green filled downward arrows point to the pH response-related residues, and brown filled downward arrows point to the functionally unrelated residues.


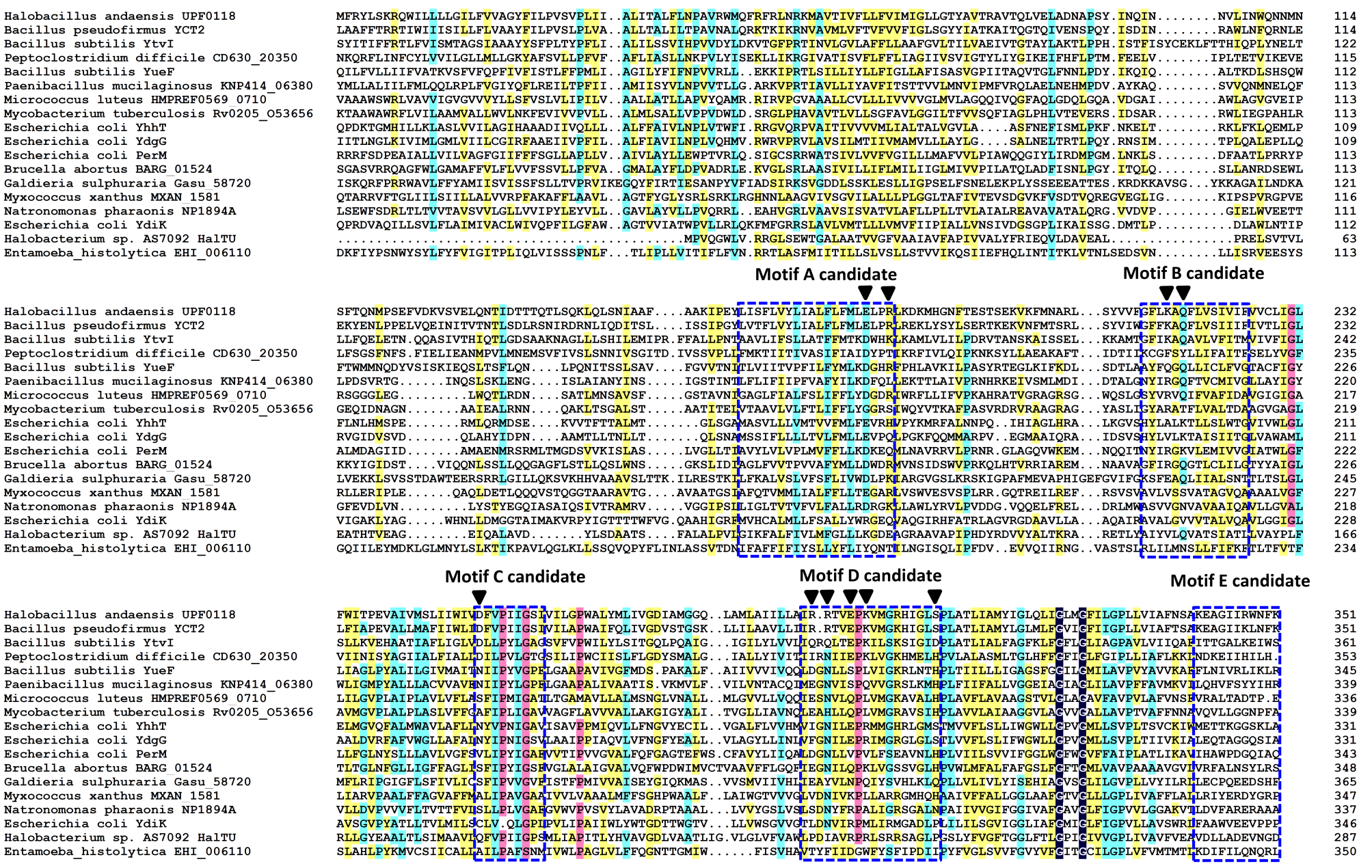


**Supplemental Figure 2. Alignment of UPF0118 with all the autoinducer-2 Exporter (AI-2E) family members collected in TCDB.**

UPF0118 was aligned with all the Autoinducer-2 Exporter (AI-2E) family members collected in TCDB. Shading homology corresponds to 100% (black), ≥ 75% (pink), ≥ 50% (cyan), ≥ 33% (yellow) and < 33% (white) amino acid identity, respectively. Five highly conserved motif (A to E) candidates of Na^+^/H^+^ Antiporter Group are highlighted within blue dash-line border open rectangles. Black filled downward arrows point to the significantly different residues between Na^+^/H^+^ Antiporter Group and other two AI-2E groups. TCDB numbers and accession numbers of the selected proteins are listed in Fig. 1.

**Supplemental Figure 3. Verification of the construct pET22b-Pro-UPF0118 based on the growth tests of *E. coli* KNabc transformants.**

For the NaCl (A) or LiCl (B) tolerance test, *E. coli* KNabc transformants were grown in the LBK media at pH 7.0, to which NaCl or LiCl was added at indicated concentrations. To test the alkaline pH (C) resistance, the same KNabc transformants were grown in LBK medium containing 50 mM NaCl at indicated pHs adjusted by adding the Hepes-Tris buffer at the final concentration of 100 mM. Each data point represents the average ± SD of three independent cultures.


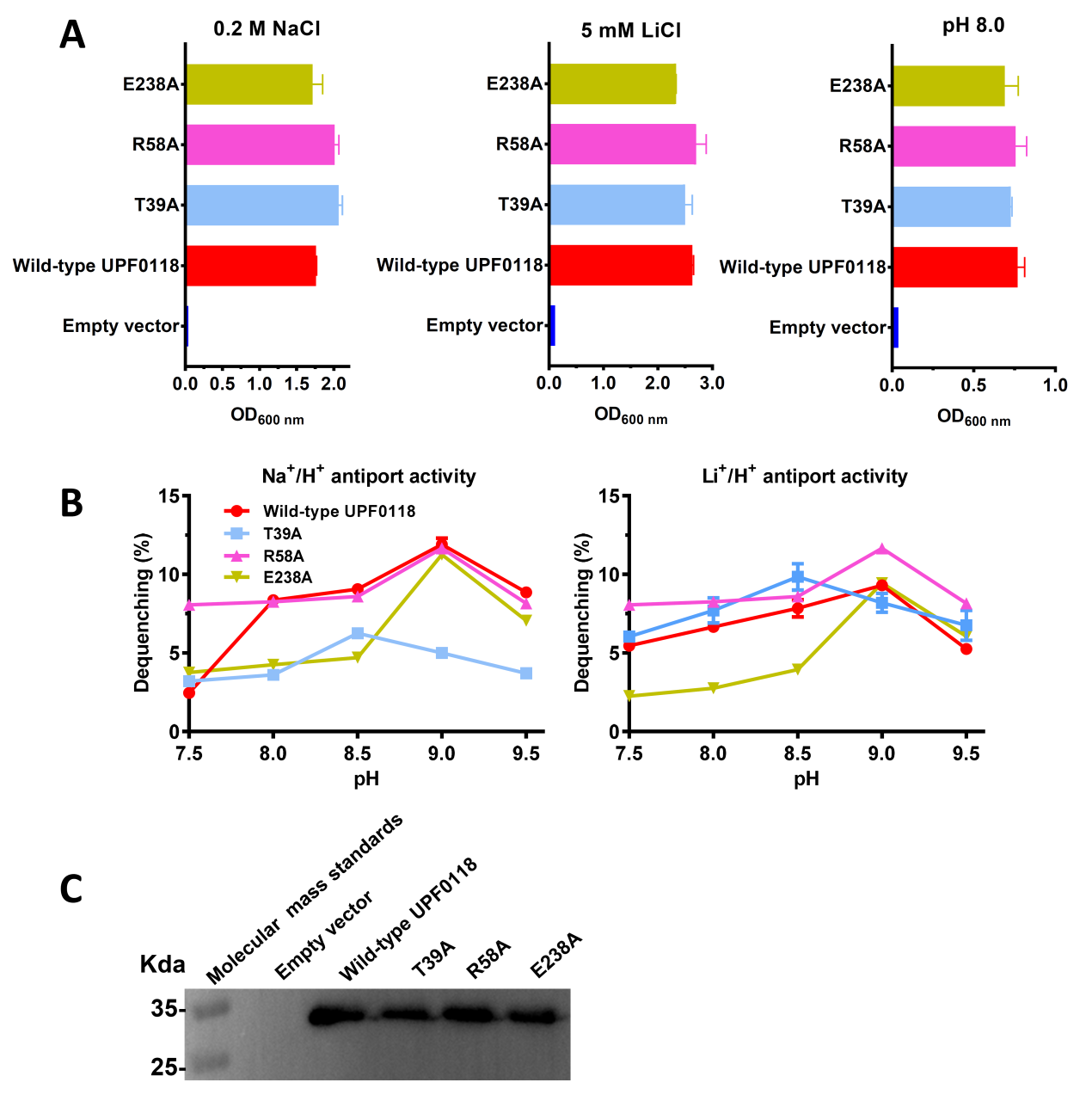


**Supplemental Figure 4. Functional analysis of the variants, T39A, R58A and E238A, based on the growth tests and antiport activity assays.**

Growth tests were carried out in the LBK media containing 0.2 M NaCl (A, left panel), 5 mM LiCl (A, middle panel) or at pH 8.0 plus 50 mM NaCl (A, right panel). Each data point represents the average ± SD of three independent cultures. Na^+^/H^+^ (B, left panel) or Li^+^/H^+^ (B, right panel) antiport activities at the pH range of 7.5 to 9.5 were analyzed by using the everted membrane vesicles from *E. coli* KNabc transformants expressing the tested variants, together with wild-type UPF0118 as the positive control. The expression levels (C) were also analyzed by using the everted membrane vesicles from the corresponding *E. coli* KNabc transformants.
